# Associations between cognitive impairment at onset and disability accrual in young people with multiple sclerosis

**DOI:** 10.1038/s41598-019-54153-7

**Published:** 2019-12-02

**Authors:** Antonio Carotenuto, Marcello Moccia, Teresa Costabile, Elisabetta Signoriello, Damiano Paolicelli, Marta Simone, Giacomo Lus, Vincenzo Brescia Morra, Roberta Lanzillo, Cogniped study group, Laura Rosa, Anna Maria Barbarulo, Fabrizia Falco, Rosa Gemma Viterbo, Francesca Lauro

**Affiliations:** 1 Department of Neurosciences, Reproductive and Odontostomatological Sciences, Federico II University, Naples, Italy; 2 Multiple Sclerosis Centre, II Division of Neurology, Department of Clinical and Experimental Medicine, ‘‘Luigi Vanvitelli” University, Naples, Italy; 3 Department of Basic Medical Sciences, Neuroscience and Sense Organs, University of Bari ‘‘Aldo Moro”, Bari, Italy; 4 Unit for Severe Disabilities in Developmental Age and Young Adults, Developmental Neurology and Neurorehabilitation, Scientific Institute IRCCS E. Medea, Brindisi, Italy

**Keywords:** Predictive markers, Cognitive neuroscience

## Abstract

Differently from the adult multiple sclerosis (MS) population, the predictive value of cognitive impairment in early-onset MS is still unknown. We aim to evaluate whether cognitive performances at disease onset predict disease progression in young people with MS. This is a retrospective study on early onset (<25 years) MS patients, who had a baseline cognitive evaluation at disease onset. Demographic and longitudinal clinical data were collected up to 7 years follow up. Cognitive abilities were assessed at baseline through the Brief Repeatable Battery. Associations between cognitive abilities and clinical outcomes (occurrence of a relapse, and 1-point EDSS progression) were evaluated with stepwise logistic and Cox regression models. We included 51 patients (26 females), with a mean age at MS onset of 17.2 ± 3.9 years, and an EDSS of 2.5 (1.0–6.0). Over the follow-up, twenty-five patients had at least one relapse, and 7 patients had 1-point EDSS progression. Relapse occurrence was associated with lower 10/36 SPART scores (HR = 0.92; p = 0.002) and higher WLG scores (HR = 1.05; p = 0.01). EDSS progression was associated with lower SDMT score (OR: 0.70; p = 0.04). Worse visual memory and attention/information processing were associated with relapses and with increased motor disability after up to 7-years follow-up. Therefor, specific cognitive subdomains might better predict clinical outcomes than the overall cognitive impairment in early-onset MS.

## Introduction

Multiple sclerosis (MS) usually commences between 18 and 50 years of age, but up to 5% of cases might experience the first symptoms even before being 18 years^1^. Early-onset MS patients have usually more pronounced inflammatory features (e.g., relapses) and take longer time to convert toward progressive MS phenotype, where disability accumulates independently from relapses^2^ but, overall, they reach the secondary-progressive MS (SP-MS) stage at a younger age (about 10 years younger than the adult counterpart)^2^.

MS-related disability arises not only from motor deficits but also from cognitive impairment, which affects approximately 30% of early-onset MS (age at onset <25 years)^3–6^, with a negative impact on everyday activities^6,7^. Processing speed and attention, visual-motor function and memory^8^ and semantic fluency^4^ are the most commonly affected cognitive domains in early-onset MS.

As cognitive impairment in MS reflects underlying inflammatory and neurodegenerative pathological features of the disorder^9^ it is highly presumptive its role in predicting the disease course over time. Cognitive impairment at disease onset is an already established biomarker of disease progression over medium and long-term follow-up in adult MS patients^10–12^. However, the predictive value of cognitive impairment for disease course in early-onset MS patients has never been evaluated. Therefore, the primary aim of this work was to evaluate whether cognitive impairment at disease onset in adolescents and young adults with MS can predict the disease burden, both in terms of relapse and disability accrual, up to seven years after disease onset.

## Methods

### Study design

This is a multi-centre retrospective study including newly diagnosed early onset relapsing-remitting (RR)-MS subjects (<=25 years), with cognitive assessment at diagnosis, that have been followed-up up to 7 years with al least 2 yearly clinical assessments in order to evaluate both the occurrence of a relapse or a sustained 1-point increase at follow-up.

### Subject enrolment

We included consecutive young subjects receiving a new diagnosis of RR-MS from January 2008 to January 2012 within specialized MS Centres at ‘Federico II’ University (Naples, Italy), ‘Luigi Vanvitelli’ University (Naples, Italy), and ‘Aldo Moro’ University (Bari, Italy). Selected centres routinely assess cognition in newly diagnosed MS patient at baseline as part of standard care. Inclusion criteria were: (i) diagnosis of RR-MS according to 2010 McDonald’s criteria^13^, (ii) age between 12 and 25 years old, (iii) no previous disease modifying treatment (DMT), and (iv) disease duration ≤6 months, (v) assessment via Brief Repeatable Battery (BRB), version A^14^. Exclusion criteria were i) medical conditions (i.e. clinical depression or psychosis) or treatments that might impact on cognitive performances (i.e. chemotherapy, or corticosteroid treatment from less than 1 month), and ii) visual or motor impairment, compromising neuropsychological performances.

Socio-demographic (age, gender and education) and clinical data (age at onset, disease duration, and EDSS) were collected at disease onset. We classified patients into paediatric (<18 years) and young adulthood onset MS (between 18 and 25 years).

MS subjects were treated with different DMTs, possibly changed or discontinued during the study period as for clinical practice. Follow-up visits were scheduled as for clinical practice (mostly every three months or on the occasion of relapses).

### Neuropsychological assessments

Cognitive function was assessed within 6 months from disease onset using BRB, version A^14^. BRB was administered to all subjects in a standardized manner, in a fixed order, and in a quiet room. The whole battery took about 30 minutes and included the following tests: the Selective Reminding Test-Long Term Storage (SRT-LTS), Selective Reminding Test-Consistent Long Term Retrieval (SRT-CLTR), and Selective Reminding Test-Delayed (SRT-D) to assess verbal memory; the 10/36 Spatial Recall Test (10/36 SPART) and 10/36 Spatial Recall Test-Delayed (10/36 SPART-D) to assess visual memory; the Symbol Digit Modalities Test (SDMT) to assess information processing speed and executive functions; the Paced Auditory Serial Addition Test 2 and 3 (PASAT 2–3) to assess attention, information processing speed, and working memory; the Word List Generation (WLG) to assess semantic fluency.

For MS patients older than 18 years, we adjusted the raw scores obtained at each test for age, gender, and/or education according to the Italian validation study in adults^14^. For patients younger than 18 years, we adjusted the raw scores obtained at each single test for age, gender, and/or education according to the Italian validation study in paediatric population^14^. We considered a test as failed when the score was at least 2 standard deviation below the mean normative values^15^. Cognitive impairment was defined as failure in at least three tests of the BRB at baseline evaluation, as from previous studies^15^.

### Clinical outcomes

During follow-up visits the following outcomes were reported:

- Occurrence of clinical relapse: we recorded the number of relapses over the follow-up period, and calculated the Annualized Relapse Rate (ARR) (number of relapses over the follow-up period); the time to first relapse from the baseline assessment was recorded (time to relapse);
- 6 months confirmed 1-point EDSS progression: we recorded both the occurrence and the time to the 1-point EDSS progression after disease onset (time to 1-point EDSS progression);

Clinical assessments were performed by the same neurologists for the whole follow up in the 3 sites (RL, ES, DP).

### Statistical analyses

Statistical analyses were performed using Stata software (version 13; StataCorp LP, College Station, TX).

Demographic, clinical and cognitive features of MS patients were presented as means, median or proportions as appropriate. In order to select covariates for statistical analyses, differences for clinical characteristics (baseline EDSS, MS centre, age at onset, disease duration, and DMT - categorized into platform or highly active DMTs) were evaluated in cognitively preserved and impaired patients with unpaired two-tailed Student t-test. We did not test differences for age and gender because normative scores were already corrected for these variables. Covariates included in statistical models were MS centre, follow-up time and first prescribed DMTs.

Backward stepwise logistic regression model (p = 0.20 as the critical value for removing the variables from the model) was used to test associations between corrected scores for each BRB test at disease onset (independent variable) and each of the two clinical variables (e.g., occurrence of relapse and 1-point EDSS progression) (dependent variables). Odds ratio (OR) and 95% confidence interval (CI) were calculated.

Backward stepwise Cox regression models (p = 0.20 as the critical value for removing the variables from the model) were also performed to calculate the Hazard ratios (HR) for both relapse occurrence and 1-point EDSS progression., Relapse occurrence and 1-point EDSS progression were used separately, as dependent variables while corrected scores for each BRB test were included as independent variable. HR and CI were calculated. A sensitivity analysis with 100-repetition bootstrap method was performed and both standard errors and CIs were calculated for both logistic and Cox regression.

Normal distribution of variables and/or residuals was evaluated through statistical and graphical approaches. Results were considered statistically significant for p < 0.05.

### Ethical standards

Since clinical and cognitive assessments were part of the clinical practice and results from the present studies did not interfere with medical decision, specific ethics approval was not required as per Italian law (G.U. No. 72 - 26^th^ March 2012). The study was performed in accordance with good clinical practices and the Declaration of Helsinki. DP, LG, VBM and RL had access to patients’ demographic features but had access only to aggregated results after statistical analysis.

## Results

### Baseline features

We included 51 MS patients (26 females and 25 males). Thirty-three patients (65%) had pediatric onset (<18 years), while 18 patients (35%) had a disease onset between 18 and 25 years old. Demographic and clinical feature of MS patients enrolled in the study are reported in Table 1. Overall follow-up was 5.52 ± 1.75 years, ranging between 2.6 and 8.3 years.

**Table 1:** Demographic and clinical features of MS patients.

Features		
Subjects	Total	51
	‘Federico II’ University, N (%)	36 (70.6%)
	‘A. Moro’ University, N (%)	9 (17.6%)
	‘L. Vanvitelli’ University, N (%)	6 (11.8%)
Sex	Male, N (%)	25 (49%)
	Female, N (%)	26 (51%)
Age at onset, mean ± SD (Range) (years)		17.2 ± 3.9 (9–25)
Disease duration, mean ± SD (Range) (months)		3.0 ± 2.9 (0–6)
EDSS, median (Range)		2.5 (1.0–6.0)
First disease modifying therapy	No DMT, N (%)	2 (3.9%)
	IFNβ, N (%)	31 (60.8%)
	Glatiramer acetate, N (%)	1 (2.0%)
	Fingolimod, N (%)	4 (7.8%)
	Natalizumab, N (%)	8 (15.7%)
	Teriflunomide, N (%)	1 (2.0%)
	Dimethyl fumarate, N (%)	2 (3.9%)
	Alemtuzumab, N (%)	2 (3.9%)
Age at onset, subgroup	Paediatric MS (<18 years), N (%)	33 (65.0%)
	Young adult MS (>18 years), N (%)	18 (35.0%)

N = Number; SD = standard deviation.

Fourteen patients (27.5%) showed cognitive impairment, according to methods definition. Adjusted scores for each BRB test and the number of MS patients failing the tests are reported in Table 2.

**Table 2:** Rao’s Brief Repeatable Battery adjusted scores in MS patients.

Test	Scores*	% of Patients failing the test
SRT-LTS, mean ± SD	45.62 ± 13.02	16%
SRT-CLTR, mean ± SD	35.32 ± 14.31	18%
SRT-D, mean ± SD	8.63 ± 2.47	14%
10/36 SPART, mean ± SD	15.78 ± 8.42	46%
10/36 SPART-D, mean ± SD	52.98 ± 3.43	30%
SDMT, mean ± SD	52.41 ± 14.65	16%
PASAT3, mean ± SD	40.86 ± 12.53	16%
PASAT2, mean ± SD	33.39 ± 11.87	9%
WLG, mean ± SD	22.58 ± 9.12	26%

*Adjusted for age, gender and education as appropriate.

SD = standard deviation; SRT-LTS = Selective Reminding Test-Long Term Storage; SRT-CLTR = Selective Reminding Test-Consistent Long Term Retrieval; SRT-D = Selective Reminding Test-Delayed; 10/36 SPART = 10/36 Spatial Recall Test; 10/36 SPART-D = 10/36 Spatial Recall Test-Delayed; SDMT = Symbol Digit Modalities Test; PASAT 2–3 = Paced Auditory Serial Addition Test 2 and 3; WLG = Word List Generation.

After MS diagnosis, thirty-three MS patients (64.7%) received a DMT and switched to different DMT after a mean of 39 ± 20 months. Twenty MS patients (61%) switched for clinical or radiological activity, 8 MS patients (21%) switched for adverse events, and 5 patients (15%) switched for personal choice.

### Occurrence of relapse

**Figure 1:**
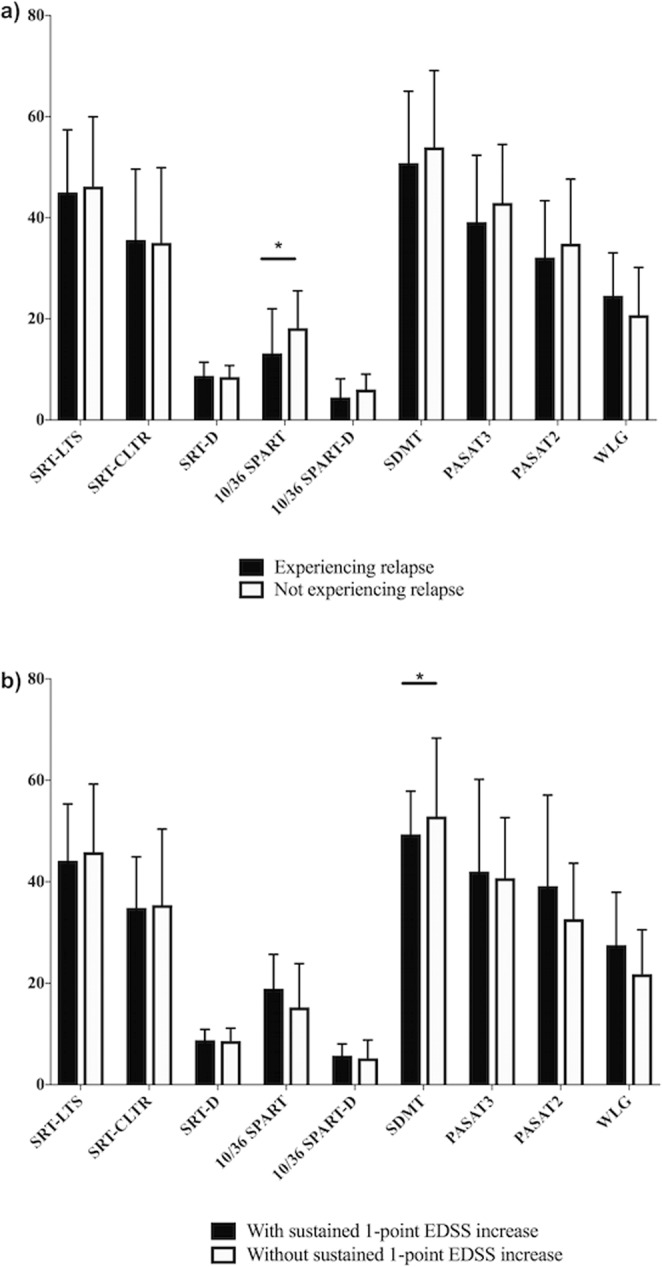
Scores on Rao’s Brief Repeatable Battery tests and clinical course over the follow-up period. (**a**) Bar graphs show mean scores for each BRB test (and standard deviation) for MS patients experiencing or not experiencing a relapse over the follow-up. The occurrence of relapse during the follow-up was associated with lower score to the 10/36 SPART (OR: 0.85, *p = 0.01). (**b**) Bar graphs show mean scores for each BRB test (and standard deviation) for MS patients experiencing or not experiencing 1-point EDSS progression, sustained over 6 months. 1-point EDSS progression was associated with lower score to the SDMT (OR: 0.70, *p = 0.04).

Twenty-five patients (49%) had at least one relapse (mean time to first relapse was 17 ± 12 months). The annualized relapse rate during follow-up was 0.35 ± 0.34. The occurrence of relapses during follow-up was associated with a lower score at 10/36 SPART (OR: 0.85; 95%CI = 0.75, 0.97, p = 0.01; see Fig. 1a). This finding was confirmed after bootstrap analysis (Bootstrap standard error: 0.05, 95%CI = 0.75, 0.95, p = 0.005).

**Figure 2:**
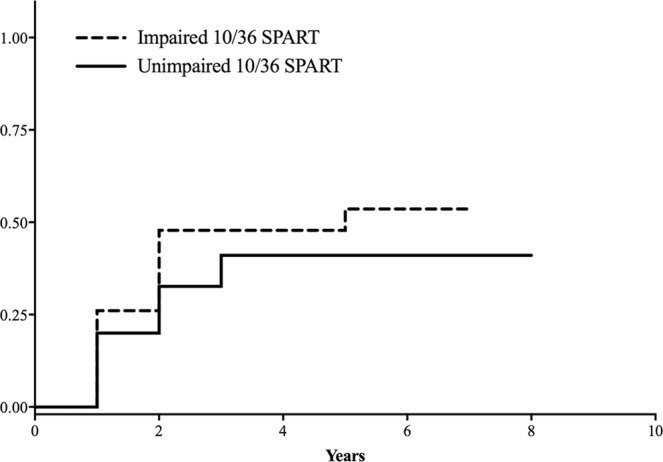
Kaplan–Meier curves for relapse occurrence and the presence/absence of cognitive deficits in selected Rao’s Brief Repeatable Battery tests. Kaplan–Meier curves for the probability of clinical relapse in relation to the presence or absence of cognitive deficit in 10/36 SPART (HR = 0.92; 95%CI = 0.88–0.97, *p = 0.002).

Higher scores at WLG and worse performances on 10/36 SPART (HR = 1.05; 95%CI = 1.01, 1.09, p = 0.01 and HR = 0.92; 95%CI = 0.88, 0.97, p < 0.01, respectively) predicted the relapse risk. However, only the predictive role of 10/36 SPART for relapse occurrence was confirmed by bootstrap analysis (Bootstrap standard error: 0.03, 95%CI = 0.86, 0.99, p = 0.03; Fig. 2).

### EDSS progression

Seven MS patients (13.7%) had 1-point EDSS progression (after 31 ± 10 months). 1-point EDSS progression was associated with a lower SDMT score (OR: 0.70; 95%CI = 0.49, 0.99, p = 0.04, Fig. 1b), and a trend towards higher 10/36 SPART score (OR: 1.67; 95%CI = 1.01, 2.75, p = 0.05). After bootstrap analysis only lower SDMT scores were associated with a higher risk of disability accrual (Bootstrap standard error: 0.11, 95%CI = 0.51, 0.95, p = 0.02).

Scores on each BRB did not predict the risk of sustained 1-point EDSS progression at Cox analysis.

## Discussion

In this retrospective multi-center study including RR-MS subjects with an early onset of disease, we investigated the presence of cognitive predictors of MS disability accrual in the medium term (up to 7 years). We found that worse cognitive function in specific domains at disease onset was associated with the risk of relapses and of sustained disability progression up to 7 years in early-onset MS. Specifically, we reported that worse visual memory and attention/information processing were associated with the occurrence of a new relapses and with increased physical disability, respectively.

The main novelty of the present study was the analysis of the predictive clinical value of cognitive impairment at disease onset in young MS patients. Clinical features suggestive of more aggressive disease course are important in driving appropriate therapeutic strategies. In adult MS patients, processing speed, verbal and spatial memory predicted motor disability over a medium-to-long-term follow-up (about 10 years)^10,11^. We partly reproduced similar results for early-onset MS. On the one hand, we observed that the performance on attention and processing speed, assessed with the SDMT, predicted the accumulation of motor disability over a medium-term follow-up, confirming previous cross sectional studies^16,17^. Moreover, information processing speed is associated with deep gray matter atrophy, especially in the thalamus, and with disconnection between subcortical and cortical networks involved in complex cognitive tasks^16^. Consistently, progressive MS patients, who show more pronounced neurodegenerative processes (e.g., brain atrophy)^18^, performed worse than RR-MS on SDMT. Noteworthy, this difference was no longer observed after controlling for the disability level^19^, suggesting that chronic neurodegenerative changes are the main driver of impaired information processing speed. Therefore, considering that early-onset MS patients have reduced grey matter volume over two years from onset^20^, which is in turn related both to SDMT and EDSS, it is not surprising that performance on SDMT at disease onset might predict the burden of motor disability over time in early-onset MS.

On the other hand, we observed that the occurrence of a new relapse over the follow-up was predicted by a lower performance in the visual memory task. This finding was not completely unexpected since visual memory deficit was already associated to the higher risk of relapsing over the follow-up in adult MS patients^10^. 10/36 SPART test requires patients to retain the pattern of randomly placed checkers on a checkerboard and further elaborate on this memory by coordinating multiple tasks, adopting advantageous retrieval strategies (e.g., focusing on one single stimulus, avoiding the intrusion of disrupting outer stimuli and manipulating information from long-term memory). Lower 10/36 SPART scores were previously observed in secondary progressive MS patients compared with primary progressive MS with similar disability levels, suggesting that visual memory performance is associated with inflammation more than neurodegeneration^21^. Supporting this hypothesis, a previous study reported an association between higher blood level of pro-inflammatory cytokines and lower scores on verbal and visual memory functions^19^. We might speculate that patients with a higher inflammatory pathology and, hence, higher probability of experiencing relapse over the follow-up, perform worse on visual memory tests at baseline.

We reported a prevalence of 27.5% of cognitive impairment in early-onset MS adopting the normative value for pediatric MS^15^ in MS patients aged less than 18 years old and adult normative value for patients aged between 18 and 25 years old. Prevalence for cognitive impairment in early-onset MS patients is generally consistent ranging from 29 to 37%, although there are variations in the definition of impairment, comparison groups (i.e., published normative values or matched groups of healthy controls) and neuropsychological battery used^4,5,22–24^. The lower prevalence we found in our sample might be due to a very short disease duration at cognitive assessment (<6 months). Previous studies usually assessed patients with a mean disease duration ranging from 1.5 to 4 years. Therefore, considering that cognitive abilities decline with disease duration^25^, our results might be in line with previous finding. However, it is also worthy to mention that previous papers usually referred to a healthy control population to define cognitive impairment, which limits the clinical applicability of cognitive evaluation. To overcome this limitation, Lanzillo and colleagues recently validated normative values of the Italian BRB in a pediatric population^15^ and, based on this, suggested that cognitive impairment definition would require the coexisting evidence of deficit in at least two tests of the BRB^14^. With this definition, prevalence of cognitive impairment in early-onset MS was 61%, whereas, with a more strict approach (setting the number of tests that must be failed for a diagnosis of cognitive impairment to three), they reported a 17% prevalence of cognitive impairment. Consequently, we applied for our sample the normative data for the BRB in patients younger than 18 years-old to define the impairment in each of the BRB test considering cognitively impaired patients failing at least three tests. Therefore, our methodology might also have impacted the overall prevalence for cognitive impairment.

Our early-onset MS population mostly failed 10/36 SPART, 10/36 SPART-D (assessing spatial memory), and WLG (assessing semantic fluency). On the contrary, adult MS patients mostly suffer from deficits in attention, information processing, verbal and spatial memory^6^. As such, early-onset MS patients displayed more severe involvement of semantic fluency, as for previous reports^26,27^. Semantic fluency relies on vocabulary funds, speed of processing and frontal functions. In healthy controls, performance in semantic fluency tests is known to steeply increase up to 23 years old, when the plateau is reached due to the progressive expansion of vocabulary funds and the improvement in controlled search through these funds^7^. The high prevalence of WLG deficits in early-onset MS might be associated with a disruption in the normal developmental process of the brain structures underpinning semantic fluency, as a consequence of demyelination/axonal loss. Moreover, early-onset MS patients could have reduced social interactions, which, in turn, results in poorer vocabulary funds.

Our study suffers from several limitations, the main one being small sample size, due to the rarity of pediatric onset MS and the lack of homogeneity of cognitive evolution in different centers, necessary to be included in the study. Moreover we analyzed the data retrospectively, although they were collected in a prospective way, leading to possible selection bias. Moreover, the retrospective nature of the study does not allow to completely rule out that different clinical features at baseline evaluation between groups with or without disease activity over the follow-up, both in terms of clinical relapse or disability worsening, might have affected our analysis. However, the long-term follow-up and appropriate statistical methods were applied to reduce these possible confounding factors. Eventually, we applied the BRB, while a more extensive neuropsychological evaluation, i.e. including phonemic fluency, emotion recognition and theory of mind, could have more clearly defined subdomains of cognitive dysfunction and their clinical correlates.

In conclusion, whilst the role of cognitive impairment in adult MS patients in predicting clinical outcomes is already well-established, we suggest that the impairment in selected cognitive domains might predict relapse risk and disability progression in early-onset MS. Although our results are preliminary and deserve further confirmations, the detection of such impairment might lead clinicians towards more active treatments since the very early stages of the disease, also in early-onset MS, avoiding relapses and irreversible disability accrual.
